# Early oocyte triggering followed by in vitro maturation is a good approach in women with resistance ovary syndrome: A case-series

**DOI:** 10.18502/ijrm.v19i6.9378

**Published:** 2021-07-27

**Authors:** Maryam Eftekhar, Banafsheh Mohammadi, Nasim Tabibnejad, Mohammad Hossein Razi

**Affiliations:** Research and Clinical Center for Infertility, Yazd Reproductive Sciences Institute, Shahid Sadoughi University of Medical Sciences, Yazd, Iran.

**Keywords:** Arrested stimulation cycle, Early oocyte triggering, In vitro maturation, Clinical outcome.

## Abstract

**Background:**

Some women represent the inability to respond to endogenous and exogenous gonadotropins during in vitro fertilization/intracytoplasmic sperm injection cycles leading to the follicular developmental arrest. The women with resistant ovaries could benefit from in vitro maturation.

**Case:**

This case-series presents pregnancies resulting from initially scheduled conventional in vitro fertilization which led to arrested cycles because of resistant ovary syndrome. The protocol was changed to early oocyte triggering for 15 women due to the small follicles ≤ 12 mm in diameter on day 15 after stimulation with high doses of exogenous gonadotrophins instead of cycle cancellation. Germinal vesicle and metaphase I oocytes that were retrieved from follicles were matured in vitro and inseminated by intracytoplasmic sperm injection. Twenty formed embryos were transferred on day 3 after oocyte retrieval. This resulted in a 30.76% chemical pregnancy out of which no abortion occurred. Therefore, we reported a 30.76% singleton ongoing pregnancy.

**Conclusion:**

It seems that early oocyte triggering followed by in vitro maturation may be considered as a good modality in women experiencing follicular resistance to gonadotropins. These cycles can be rescued from cancellation with satisfactory clinical outcomes.

## 1. Introduction

The administration of gonadotropin to stimulate multiple follicular developments and the following transfer of good-quality embryos has intensely improved the success of assisted reproductive technologies.

The good ovarian response to gonadotropins with sufficient follicular development is necessary for a successful assisted reproductive technologies cycle. However, some women represent the inability to respond to endogenous and exogenous follicle-stimulating hormone (FSH) leading to the follicular developmental arrest (1–3).

Women with resistant ovaries show slow-growing follicles, however, they usually present normal follicle numbers, but the follicles size is likely ≤ 12 mm in diameter on day 15 of the treatment cycle. An extended stimulation period and a higher dose of gonadotropins may be helpful in these women, but they experience a high rate of cycle cancelation due to immature follicles (4, 5).

With the introduction of in vitro maturation (IVM), women with inadequate response to ovarian stimulation during in vitro fertilization (IVF) cycles could benefit from this technique. During the IVM procedure, the immature oocytes following their recovery from small follicles develop from germinal vesicle (GV) in prophase meiosis I (MI) or those in metaphase MI to mature metaphase II (MII) in the culture media (6).

There are still controversies on the efficacy of IVM as a beneficial option in poor responders or those with delayed follicular development. It is expected that the early triggering of follicles < 12 mm result in a mixed pool of in vivo-matured MII, MI, and GV stage oocytes that may disturb routine IVM culture and intracytoplasmic sperm injection (ICSI) timing for the entire oocyte collection (7). However, we assumed that this method may be a cost-benefit approach in these poor-prognosis patients rather than cycle cancellation, which has undesired psychological and financial problems.

Here, we present our experience with the efficiency of early oocyte triggering and immature oocyte retrieval followed by IVM in cases of poor response to high doses of exogenous gonadotropins during an IVF treatment regimen.

## 2. Case Presentation

In this prospective case-series, we studied 15 cases undergoing ICSI who presented good ovarian reserve but insufficient ovarian response to ovarian stimulation from August 2019 to April 2020 at the Yazd Reproductive Sciences Institute, Yazd, Iran. All women had normal FSH with a normal antral follicle count (AFC) ≥ 12 and normal or high serum anti-Müllerian hormone (AMH) > 1.1 ng/mL. Women with diminished ovarian reserve and abnormal semen parameters of the male partner were excluded.

These women had been initially scheduled for routine IVF procedure but their protocol was changed due to the arrested cycles. They planned to undergo an early oocyte triggering and IVM program instead of cycle cancelation.

At first, women had been stimulated using a gonadotropin-antagonist protocol. For this purpose, they received Cinnal f (Follitropin alfa, Cinnagen, Tehran, Iran) 150 IU subcutaneously once a day starting the second day of stimulation. Follicular monitoring was performed following the ovarian stimulation on day 6 of the cycles. However, due to the arrested follicular development, gonadotropin-releasing hormone antagonists were not administered. Gonadotropin therapy was continued until the 15${}^{\mathrm{th}}$ day of their cycles. No case of ovarian hyperstimulation syndrome occurred.

Because of the follicular arrest and no presence of any dominant follicle > 12 mm, women were offered to change their treatment protocols to early oocyte triggering followed by IVM rather than cycle cancellation. Oocytes of women who accepted the clinician's suggestion were retrieved. Oocyte retrieval was performed 36 hr after an intramuscular injection of 10,000 IU human chorionic gonadotropin (hCG) (Pregnyl, Organon, Netherlands) by a 17-gauge aspiration needle using transvaginal ultrasound guidance with a reduced aspiration pressure of 100 mmHg. Immature oocytes were transferred to IVM culture medium (SAGE Co., CT, USA), supplemented with 75 mIU/mL FSH and 75 mIU/mL luteinizing hormone (LH) (Ferring GmbH, Kiel, Germany), and 20% human serum albumin (HSA) at a temperature of 37°C in an atmosphere of 5% CO2 with high humidity for 24 hr without medium renewal. Oocyte maturation was evaluated by the detection of the first polar body under a stereomicroscope (Nikon, Japan).

The mature oocytes were injected using husbands' sperm and normal fertilization was proven by observing two pronuclei 18–20 hr following the insemination.

Up to two good-quality embryos were transferred two days after the microinjection using an embryo transfer catheter (Labotect, Gottingen, Germany). Luteal phase support was performed with CyclogestⓇ vaginal suppository (Cox Pharmaceuticals, Barnstaple, UK), 400 mg twice a day. Estradiol valerate (Aburaihan Co., Tehran, Iran) was administered orally in the dose of 6 mg/day from the day of oocytes retrieval and continued until observation of the fetal heart through ultrasonography. βhCG was measured 14 days after the embryo transfer for detecting chemical pregnancy, and if it remained positive until 12${}^{\mathrm{th}}$ wk of pregnancy, it was considered a normal ongoing pregnancy. Women were followed-up until the 20${}^{\mathrm{th}}$ wk of pregnancy.

The clinical characteristics of all patients are listed in Table I. The patients' mean age was 29.33 ± 4.06 yr and their mean body mass index was 24.79 ± 1.97. The mean duration of infertility was 5.66 ± 3.73 yr among the patients. The mean AMH level was 6.74 ng/mL with a maximum of 16.80 and a minimum of 1.70 ng/mL. The mean endometrial thickness was 9.33 mm. Thirteen GV-stage, nine MI-stage, and 28 in-vivo matured MII-stage oocytes were collected. Ten out of 13 GV oocytes and all nine MI oocytes reached MII.

All in-vivo and in-vitro matured MII oocytes were inseminated by ICSI. In two cycles no embryo was formed. In total, 28 good-quality embryos were obtained, of which, 20 were transferred freshly and 8 were vitrified. Embryo transfers resulted in 4/13 (30.76%) chemical pregnancy, out of which no abortion or ectopic pregnancy occurred. Therefore, we had a 30.76% singleton ongoing pregnancy.

**Table 1:** Clinical characteristics of all patients


**Variable**	**Case number**														
	**1**	**2**	**3**	**4**	**5**	**6**	**7**	**8**	**9**	**10**	**11**	**12**	**13**	**14**	**15**
**Age (yr)**	28	30	29	29	24	27	29	32	28	27	22	32	29	36	38
**Duration of infertility (yr)**	4	7	3	9	3	3	4	3	4	5	2	7	12	4	15
**BMI (kg/m ${}^{2}$)**	23	22	27	23	21	25	23	26	26	25	24	26	22	25	28
**AMH (ng/mL)**	10.8	10.0	16.8	10.8	9.4	5.9	3.7	7.4	4.8	2.5	4.1	1.7	5.1	4.9	3.1
**Endometrial thickness (mm)**	9.8	10.0	8.5	10.0	8.6	7.5	11.0	7.8	8.6	12.0	7.5	8.3	8.8	9.6	12.0
**No. of gonadotropin ampules**	26	38	28	26	30	28	34	24	34	32	41	56	40	30	50
**Duration of gonadotropin administration (day)**	15	16	15	17	16	15	17	19	16	15	17	17	15	16	15
**No. of GV oocytes retrieved**	3	3	1	–	–	1	–	–	1	1	–	–	–	3	–
**No. of MI oocytes retrieved**	2	3	–	–	–	–	–	2	–	–	–	–	–	–	2
**No. of in-vivo matured MII oocytes**	–	–	–	6	2	2	1	–	–	4	1	4	2	–	6
**No. of MII oocyte after IVM**	3	5	1	–	–	1	–	2	1	1	–	–	–	3	2
**No. of embryo formed**	3	4	1	2	1	1	1	2	1	4	–	2	1	–	5
**No. of embryo transferred**	2	2	1	2	1	1	1	2	1	2	–	2	1	–	2
**Embryo quality**	B	A	B	B	B	A	A	B	B	A	–	A	B	–	A
**Outcome**	βhCG-	βhCG-	βhCG-	βhCG-	OP	OP	βhCG-	βhCG-	OP	βhCG-	–	OP	βhCG-	–	βhCG-
BMI: Body mass index, AMH: Anti-Mullerian hormone, GV: Germinal vesicle, MI: Metaphase I oocyte, MII: Metaphase II oocyte, IVM: In vitro maturation, βhCG: βHuman chorionic gonadotropin, OP: Ongoing pregnancy															

### Ethical considerations

The study protocol was approved by the Ethics Committee of Yazd Reproductive Sciences Institute, Shahid Sadoughi University of Medical Sciences (Code: IR.SSU.RSI.REC.1397.029).

All participants signed a written informed consent for their information to be included in the study. Confidentiality was provided by participant de-identification.

## 3. Discussion

This case series reported the reasonable percentages of ongoing pregnancies resulting from the trigger of small follicles in arrested cycles. The present study indicated that early oocyte triggering followed by IVM in stimulated antagonist cycles may be a good alternative with satisfactory outcomes in women with good ovarian reserve whose follicles poorly responded to gonadotropin stimulation.

In the current study, we reported 15 cases with poor response to ovarian stimulation. Similar to our work, Hatirnaz and colleagues reviewed records of 13 patients with follicular arrest ranging from poor to hyper responders. IVM was applied as a rescue procedure and antagonist-stimulated IVF cycles were shifted to IVM. Six pregnancies (46.1%) and four healthy live births were reported (8). Likewise, Li and colleagues reported a live birth in a 33-yr-old woman with resistant ovary syndrome. The patient had oligomenorrhea and increased gonadotropin levels with normal AFC. After the administration of high doses exogenous gonadotropins, five immature oocytes were retrieved. Following the IVM, two top-quality embryos were transferred which led to pregnancy and delivery of a healthy baby (9). Besides, Grynberg and colleagues reported a woman with resistant small antral follicles to FSH, who achieved a live birth after rescue IVM. They aspirated 15 GV-stage oocytes, out of which 12 reached MII and were injected by ICSI (2). Moreover, a case series reported 28 cases of resistant ovary syndrome and incomplete oocyte maturation. Among the nine patients with normal AFC who were resistant to ovarian stimulation, IVM followed by ICSI resulted in a live birth rate of 33.3% per patient. The other 19 women with a history of poor oocyte maturation underwent IVM but no live birth was achieved in this group. The authors concluded that IVM is a practical method in patients with resistant ovary syndrome, but not in patients with deficient oocyte maturation (10). In all the aforementioned studies, only premature oocytes in the GV and MI stages of nuclear maturity were retrieved. Grynberg et al. and also Li and colleagues reported the oocyte maturation rates of 80% and 60%, respectively (2, 9). However, Galvao and colleagues stated a low maturation rate of 29.7%, they explained that this difference is due to the absence of hCG oocyte-triggered cycles (10).

Only one study like ours retrieved a collection of oocytes in the different nuclear maturity status. They reported a case of polycystic ovary syndrome that received gonadotropin for 3–5 days and then triggered using GnRH antagonist when the largest follicle was 12 mm in diameter. All in-vivo or in-vitro-matured MII oocytes were inseminated and following embryo transfer resulted in a live birth (11).

All above-mentioned successful work showed that the trigger of small follicles resistant to gonadotropin may be a helpful option for the patient at risk for cycle cancelation. Indeed, the subcategory of infertile patients with a normal ovarian reserve and inadequate response to ovarian stimulation is considered as a major challenge for fertility clinics (10).

These patients and the IVF team have to bear the financial and psychological burden due to the cycle cancellation or cycle extension. On the other hand, the mechanisms of ovarian stimulation failure in these patients including follicular developmental arrest or delayed oocyte maturation are not known.

Therefore, an accurate diagnosis cannot be made in most of these patients and they often undergo several ineffective treatment attempts. Nevertheless, based on the aforementioned studies, it seems that the acceptable pregnancy rate could be obtained in early oocyte retrieval and consequent IVM cycles. In fact, this rescue procedure may be effective to retrieve even one more oocyte from the few remaining oocytes in these poor prognosis patients, because the achievement of one additional embryo for transfer could increase the chances of pregnancy as well as decreasing the probability of cycle cancelation.

## 4. Conclusion

In conclusion, it seems that early oocyte triggering followed by IVM may be considered as a good modality in women experiencing follicular resistance to routine ovarian stimulation protocols. These cycles can be rescued from cancellation with satisfactory clinical outcomes.

##  Conflict of Interest

The authors have no conflicts of interest to declare.
